# The Role of Hub and Spoke Regions in Theory of Mind in Early Alzheimer’s Disease and Frontotemporal Dementia

**DOI:** 10.3390/biomedicines10030544

**Published:** 2022-02-24

**Authors:** Beatrice Orso, Luigi Lorenzini, Dario Arnaldi, Nicola Girtler, Andrea Brugnolo, Elisa Doglione, Pietro Mattioli, Erica Biassoni, Federico Massa, Enrico Peira, Matteo Bauckneht, Maria I. Donegani, Silvia Morbelli, Flavio Nobili, Matteo Pardini

**Affiliations:** 1 Department of Neuroscience, Rehabilitation, Ophthalmology, Genetics, Maternal and Child Health (DINOGMI), University of Genoa, Largo Daneo 3, 16132 Genoa, Italy; beatrice.orso@edu.unige.it (B.O.); dario.arnaldi@unige.it (D.A.); nicolagirtler@unige.it (N.G.); andrea.brugnolo@unige.it (A.B.); s3636680@studenti.unige.it (P.M.); s3773837@studenti.unige.it (E.B.); 3105049@studenti.unige.it (F.M.); matteo.pardini@unige.it (M.P.); 2 Department of Radiology and Nuclear Medicine, Amsterdam UMC Location VuMC, Amsterdam Neuroscience, Vrije Universiteit Amsterdam, 1081 HV Amsterdam, The Netherlands; l.lorenzini@amsterdamumc.nl; 3 IRCCS Ospedale Policlinico S. Martino, Largo Rosanna Benzi 10, 16132 Genoa, Italy; 2932295@studenti.unige.it (E.D.); matteo.bauckneht@hsanmartino.it (M.B.); isabella.donegani@gmail.com (M.I.D.); silviadaniela.morbelli@hsanmartino.it (S.M.); 4 National Institute of Nuclear Physics (INFN), Genoa Section, Via Dodecaneso 33, 16146 Genoa, Italy; epeira@ge.infn.it; 5 Department of Health Science (DISSAL), University of Genoa, Via Antonio Pastore 1, 16132 Genoa, Italy

**Keywords:** Alzheimer’s disease, frontotemporal dementia, theory of mind, brain network, ^18^F-FDG-PET, social cognition, neuroimaging, neurodegenerative diseases, dementia, mild cognitive impairment

## Abstract

Theory of mind (ToM, the ability to attribute mental states to others) deficit is a frequent finding in neurodegenerative conditions, mediated by a diffuse brain network confirmed by ^18^F-FDG-PET and MR imaging, involving frontal, temporal and parietal areas. However, the role of hubs and spokes network regions in ToM performance, and their respective damage, is still unclear. To study this mechanism, we combined ToM testing with brain ^18^F-FDG-PET imaging in 25 subjects with mild cognitive impairment due to Alzheimer’s disease (MCI–AD), 24 subjects with the behavioral variant of frontotemporal dementia (bvFTD) and 40 controls. Regions included in the ToM network were divided into hubs and spokes based on their structural connectivity and distribution of hypometabolism. The hubs of the ToM network were identified in frontal regions in both bvFTD and MCI–AD patients. A mediation analysis revealed that the impact of spokes damage on ToM performance was mediated by the integrity of hubs (*p* < 0.001), while the impact of hubs damage on ToM performance was independent from the integrity of spokes (*p* < 0.001). Our findings support the theory that a key role is played by the hubs in ToM deficits, suggesting that hubs could represent a final common pathway leading from the damage of spoke regions to clinical deficits.

## 1. Introduction

Theory of Mind (ToM) is the ability to attribute mental states to others and to predict, describe and explain behavior on the basis of such mental states [1]. ToM deficits are clinically relevant for their impact on daily activities and in some cases, they may represent one of the earliest signs of cognitive decline, as seen in different neurodegenerative diseases [2,3].

ToM is thought to be mediated by a distributed cortical network of frontal and posterior cortical regions, including the middle prefrontal cortex (mPFC), the inferior parietal lobe, the posterior and anterior cingulate cortex (PCC, ACC), as well as the temporoparietal junction (TPJ), the amygdala and the superior temporal sulcus (STS) [4,5,6].

An open question remains regarding the relative importance of each of these regions in leading to ToM deficits across different clinical conditions and the ability of functional imaging to capture those changes more relevant to ToM performance in the earliest phases of the neurodegenerative process. Studies using brain ^18^F-fluorodeoxyglucose positron emission tomography (^18^F-FDG-PET) and tau markers have shown a negative correlation between tau deposition and cerebral metabolism in the right temporal, parietal and frontal lobes in a cohort of 100 Alzheimer’s disease (AD) patients, confirming the clinical relevance of these regions in AD and suggesting that functional imaging can capture degeneration in these areas in AD [7,8]. Moreover, functional magnetic resonance imaging (fMRI) studies on functional connectivity [9] have provided evidence of the existence of brain networks engaged in different cognitive function, such as the default mode network (DMN), comprehensive of the mPFC and PCC [10], associated with internal processing (i.e., self-referential thinking or thinking about the future) [11]; the executive control network (ECN), of which changes have been reported in mild cognitive impairment (MCI) [12], AD [13] and non-demented patients with late-life depression [14]; as well as the salience network (SN), responsible for detecting and incorporating sensory and emotional stimuli, both in healthy subjects and in neuropsychiatric conditions, which is located in the ventral and anterior insula and includes the amygdala, thalamus, striatum and hypothalamus [15,16]. Lastly, a recent study from Battaglia et al. (2021) [17] showed that the impairment of the (ventromedial prefrontal cortex) vmPFC, possibly through the modulation of temporal pole regions, has an impact on the recognition of negative emotions such as fear [17]. These findings provide converging evidence of the role of networks as the key underpinning of the cognitive and affective components of ToM.

Inside the connectionist paradigm [18], it has been proposed that regions that are more connected with the other components of a network (i.e., the so-called “hubs”) are more relevant to network functioning than those less connected (i.e., the “spokes”) [19]; however, their relative roles (and their interactions) in clinically relevant deficits are only partly understood. In AD, for example, hubs are thought to present structural damage early on in the disease course, possibly due to an increase in oxidative stress in these regions [20]. In subjects with stroke, worse cognitive outcomes have also been associated with structural damage to hubs [21].

Given these observations, we decided to evaluate the relative clinical relevance of the regions included in the ToM network, a priori chosen, taking into account their structural connectivity and the extent of local neurodegeneration, measured using brain ^18^F-FDG-PET. To this aim, we focused on mild cognitive impairment due to AD (MCI–AD) and on behavioral variant frontotemporal dementia (bvFTD) patients, given the differences in the neurodegeneration distribution pattern between these two conditions. To go into more detail, combining a widely used ToM test (i.e., the Reading the Mind in the Eyes test—RMET) and brain ^18^F-FDG-PET, we evaluated whether hub regions played a more significant role on ToM performance than spoke regions, and if the impact of spoke regions damage on ToM performance was mediated by the extent of degeneration in the hubs.

## 2. Materials and Methods

### 2.1. Participants

We enrolled twenty-five consecutive patients (9 males; mean age 80.72 ± 5.61) affected with intermediate-likelihood mild cognitive impairment due to AD (MCI–AD), according to the NIA-AA criteria [22,23], and twenty-four consecutive patients (6 males; mean age 75.58 ± 7.66) with mild, behavioral frontotemporal dementia (bvFTD) [24]. All subjects fulfilled the following inclusion criteria:

(i) MMSE score ≥26 and CDR score ≤1 at the time of enrollment;
(ii) Symptoms duration less than two years at enrollment;
(iii) Confirmation of clinical diagnosis after at least two years of follow-up;
(iv) No current or past diagnosis of a comorbid psychiatric or neurological condition, or other major medical conditions;
(v) No regular use of psychoactive drugs;
(vi) A brain ^18^F-FDG-PET scan performed no later than three months after neuropsychological testing.

Lastly, 40 healthy controls (HC) who underwent ^18^F-FDG-PET imaging in the frame of previous studies were included in this work (mean age 78.5 ± 9.4) to be used as a control group in the imaging analysis. 

Demographic and clinical data of the groups are reported in Table 1.

All subjects were able to complete all study procedures.

At the time of examination, subjects provided their written consent for the use of their anonymized data for research purposes according to the protocol approved by our institutional review board. The study was conducted in accordance with the Helsinki Declaration.

### 2.2. Theory of Mind Assessment

ToM was assessed using the “Reading the Mind in the Eyes” task developed by Baron-Cohen and colleagues (2001) [25] and revised by Harkness et al. (2005) [26]. The test consists of 36 black-and-white pictures of the eye region. The subject then has to recognize the emotional state represented in the picture and choose one among four given words that depict an emotion [27].

### 2.3. ^18^F-FDG-PET Acquisition

Patients underwent brain ^18^F-FDG-PET scan, acquired according to the guidelines of the European Association of Nuclear Medicine [28].

Subjects fasted for at least six hours. Before radiopharmaceutical injection, blood glucose was checked and was <7.8 mmol/L in all cases. After a 10 min rest in a silent and obscured room, with eyes open and ears unplugged, subjects were injected with approximately 200 MBq of ^18^F-FDG via a venous cannula. They remained in the room for 30 min after the injection and then moved to the PET room where scanning started approximately 45 min after the injection and lasted 15 min. A polycarbonate head holder was used to reduce head movements during the scan. Images were acquired by means of SIEMENS Biograph 16 PET/CT equipment with a total axial field of view of 15 cm and no interplane gap space. Attenuation correction was based on CT. Images were reconstructed through an ordered subset expectation maximization algorithm, 16 subset and 6 iterations, with a reconstructed voxel size of 1.33 × 1.33 × 2.00 mm.

^18^F-FDG-PET images of patients and HC were subjected to affine and nonlinear spatial normalization into Talairach and Tournoux space using SPM12 (Wellcome Department of Cognitive Neurology, London, UK). Images were normalized in the MNI space using SPM12 “Old Normalization” utility, allowed to follow the procedure previously used for SPM5 and SPM8 (the template used in this study was validated against all these SPM versions). Coordinates of the final results are provided in the MNI space. We used the Talairach daemon tool to convert MNI coordinates to those corresponding to the Talairach atlas. All the default choices of SPM were followed except for spatial normalization. For this study, the H_2_^15^O SPM-default template was replaced by an optimized brain ^18^F-FDG-PET template as described by Della Rosa and colleagues [29]. The spatially normalized set of images were then smoothed with a 10 mm isotropic Gaussian filter to blur individual variations in gyral anatomy and to increase the signal-to-noise ratio.

### 2.4. Reading the Mind in the Eyes Task Regions of Interest

The RMET regions of interest (ROIs) were defined based on the meta-analysis published by Molenberghs et al. (2016) [30].

In the paper, the ROIs associated with RMET were described as: the left and right middle temporal gyrus, superior temporal gyrus, cingulate gyrus, superior frontal gyrus, inferior frontal gyrus, middle frontal gyrus and the left precentral gyrus.

**Figure 1:**
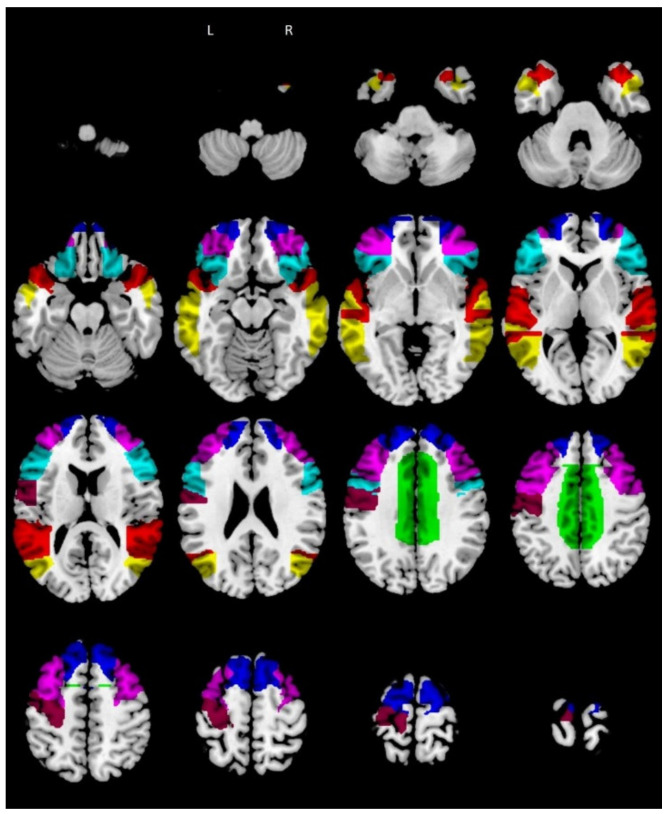
RMET ROIs according to Molenberghs et al. (2016) (RMET = Reading the Mind in the Eyes task; R = right; L = left; ROIs = regions of interest). Legend: green = R/L cingulate gyrus; cyan = R/L inferior frontal gyrus; blue = R/L superior frontal gyrus; violet = R/L middle frontal gyrus; yellow = R/L middle temporal gyrus; purple = left precentral gyrus; red = R/L superior temporal gyrus.

We created these ROIs using the WFU PickAtlas toolbox for SPM12 (Figure 1).

### 2.5. Statistical Analysis

1. Firstly, a two-sample whole-brain t-test design was performed using SPM12 (Wellcome Department of Cognitive Neurology, London, UK) between HC and both MCI–AD and bvFTD groups separately, using a height threshold *p* < 0.0001 (uncorrected) at voxel level, p family-wise error (FWE) corrected <0.05 at cluster level. Age and sex were used as nuisance variables. The resulting hypometabolic areas were then overlapped onto the RMET ROIs map (shown in Figure 1) to see if they were included in the brain metabolism distribution of RMET.

2. For the regions that showed an overlap with the hypometabolic areas within patients’ groups, we evaluated the structural connectivity profile. Specifically, we derived binarized structural connectivity matrices between RMET ROIs from the brainnetome connectivity atlas (www.brainnetome.org/, accessed on 20 May 2020) by thresholding for its maximum Shannon entropy.

3. Using the MarsBar 0.44 SPM12 toolbox we extracted the regional ^18^F-FDG-PET counts of the RMET ROIs, and we then normalized over the whole brain as follows (ROI average uptake—whole brain average uptake)/whole brain average uptake, so as to correlate them, using parametric statistics, with regional relative metabolism. Then, we used a partial correlation approach to explore the correlation between regional metabolism of the RMET ROIs and RMET performance in our patients’ groups, correcting for age, education and MMSE score.

4. Lastly, we ran a confirmatory mediation analysis with the mediation package of the R software and a bootstrap approach (1000 permutations) using hub metabolism as mediatory variable, and both spoke metabolism and RMET performance, respectively, as independent and dependent variables.

Statistical significance was set at 0.05 correcting for multiple comparisons using the false discovery rate (FDR) approach.

## 3. Results

### 3.1. Relative Hypometabolism in Behavioral Variant of Frontotemporal Dementia Patients vs. Controls

Compared to controls, bvFTD patients showed relative hypometabolism in anterior regions, including the dorsolateral and medial prefrontal cortex, the anterior and lateral temporal lobe as well as in deep gray matter regions (Supplementary Table S1).

**Figure 2:**
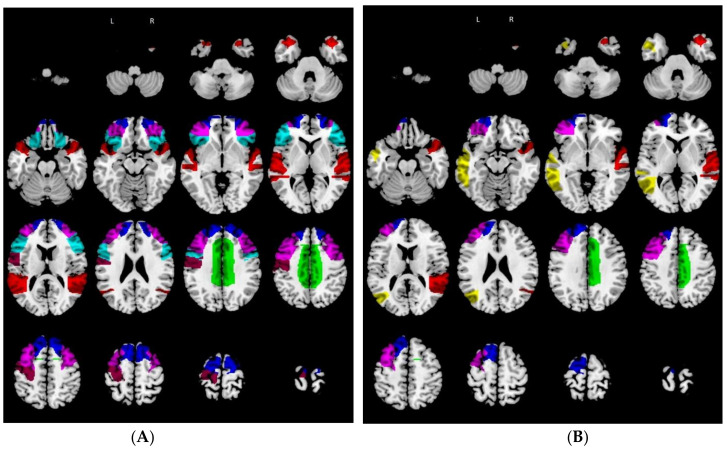
ROIs involved in the RMET network within the two patient groups. (**A**) bvFTD group. (**B**) MCI–AD group (RMET = Reading the Mind in the Eyes task; R = right; L = left; bvFTD = behavioral variant of frontotemporal dementia). Legend: green = R/L cingulate gyrus; cyan = R/L inferior frontal gyrus; blue = R/L superior frontal gyrus; violet = R/L middle frontal gyrus; yellow = R/L middle temporal gyrus; purple = left precentral gyrus; red = R/L superior temporal gyrus.

The RMET ROIs included in the areas of relative hypometabolism in the bvFTD group were the bilateral cingulate gyrus, bilateral inferior frontal gyrus, bilateral superior frontal gyrus, bilateral middle frontal gyrus, left precentral gyrus, and bilateral superior temporal gyrus (Figure 2A).

### 3.2. Relative Hypometabolism in Mild Cognitive Impairment due to Alzheimer’s Disease Patients vs. Controls

Compared to controls, MCI–AD patients showed relative hypometabolism in posterior regions, including the temporal and parietal lobes, the posterior aspect of the frontal lobe and the thalamus (Supplementary Table S2).

The RMET ROIs included in the areas of relative hypometabolism in MCI–AD group were the left cingulate gyrus, right superior frontal gyrus, right middle frontal gyrus, left middle temporal gyrus, and right superior temporal gyrus (Figure 2B).

### 3.3. Hub and Spoke Definition

From the connectivity matrices obtained as aforementioned, in the bvFTD group, the left and right superior frontal gyri presented the highest number of connections (thus representing the hubs of the network) with the other RMET ROIs (thus representing the spokes of the network) (Table 2). However, in the MCI–AD group, the left middle frontal gyrus presented the highest number of connections (thus representing the hub of the network) with the other RMET ROIs (thus representing the spokes of the network) (Table 3).

### 3.4. Correlation between Regional Metabolism of the Reading the Mind in the Eyes Task Regions of Interest and Reading the Mind in the Eyes Task Performance: Behavioral Variant of Frontotemporal Dementia

In bvFTD patients, we found a significant direct correlation between RMET performance and metabolism in the following hub ROIs: bilateral superior frontal gyrus (r = 0.72; *p* = 0.004); as well as in the following spoke ROIs: bilateral cingulate gyrus (r = 0.48; *p* = 0.033), bilateral inferior frontal gyrus (r = 0.53; *p* = 0.010), bilateral middle frontal gyrus (r = 0.65; *p* = 0.004) and left precentral gyrus (r = 0.56; *p* = 0.005).

None of the remaining ROIs presented a significant correlation with RMET performance. Results are shown in Table 4.

### 3.5. Correlation between Regional Metabolism in the Reading the Mind in the Eyes Task Regions of Interest and Reading the Mind in the Eyes Task Performance: Mild Cognitive Impairment Due to Alzheimer’s Disease

In MCI–AD patients, we found a direct correlation between RMET performance and metabolism in the following hub ROI: left middle frontal gyrus (r = 0.69; *p* = 0.01); as well as in the following spoke ROIs: left middle temporal gyrus (r = 0.43; *p* = 0.033) and left superior frontal gyrus (r = 0.50; *p* = 0.039). None of the remaining ROIs presented a significant correlation with RMET performance. Results are shown in Table 4.

### 3.6. Hub–Spoke Interactions and Reading the Mind in the Eyes Task Performance: Behavioral Variant of Frontotemporal Dementia

The correlation between average spoke metabolism and RMET performance reported above lost significance when correcting for the average metabolism in hub regions. 

This observation was confirmed by a mediation analysis, which revealed a significant full mediation of the effect on RMET score of the mean metabolism of the spoke regions by average hub metabolism (average causal mediation effects *p* < 0.001).

### 3.7. Hub–Spoke Interactions and Reading the Mind in the Eyes Task Performance: Mild Cognitive Impairment due to Alzheimer’s Disease

The correlation between average spoke metabolism and RMET performance reported above lost significance when correcting for the average metabolism in hub regions.

The observation was confirmed by a mediation analysis, which revealed a full mediation of the effect on RMET score of the spoke regions by average hub metabolism (average causal mediation effects *p* < 0.001).

## 4. Discussion

In this study, we evaluated the relative importance of the different components of a diffuse brain network to determine ToM deficits in subjects with neurodegenerative disorders. To this aim, we focused on the network sub-serving ToM in subjects with MCI–AD and bvFTD, given the spatial distribution of damage and the role played by ToM impairments in those conditions [31].

The network underlying ToM abilities is complex, and it is thought to involve primarily the limbic system, the PFC, the TPJ and the anterior and posterior cingulate cortex [4,32]. To date, however, the relative importance of each region for the development of mentalizing deficits is only partially understood.

Firstly, in preliminary analyses, we showed that ToM performance correlated with regional integrity only in those areas with a reduced ^18^F-FDG-PET metabolism in our patients’ groups compared to controls. Moreover, we showed that among those hypometabolic ROIs, ToM only correlated with frontal regions in both bvFTD and MCI–AD patients.

The involvement of frontal regions in ToM is in line with previous imaging studies and represents the neuroanatomical bases of the early development of ToM deficits in subjects with bvFTD [3] and of the later involvement of ToM in AD [33,34]. In previous studies, we showed that ToM deficits could represent the first sign of bvFTD in otherwise asymptomatic subjects [35]. Moreover, converging evidence from healthy control functional data [30] and lesion studies [31] points to a key role for the prefrontal territories in ToM. For example, Dal Monte and colleagues (2014) [36] showed an association between RMET and damage to the inferior frontal gyrus in a sample of 138 Vietnam veterans with focal brain damage, and Rowe et al. (2001) [37] reported that individuals with either right or left PFC lesions presented ToM deficits as assessed by first- and second-order false belief tests. Our results are also in line with previous ^18^F-FDG-PET studies, in which ToM has been associated with activations of prefrontal territories in a cohort of bvFTD and AD patients [38]. The involvement of frontal territories in ToM has also been shown using neurophysiological techniques such as the skin conductance response (SCR) of healthy participants [39], transcranial magnetic stimulation (TMS) [40] and rs-fMRI [41], which provide complementary and confirmatory evidence of the role of frontal circuits on cognitive functioning, depression [42] and social cognition [43]. The impact of the prefrontal cortex in ToM is also supported by the presence of prefrontal abnormalities both in autism and schizophrenia [44,45], as well as by the impact of prefrontal alterations on cognition in Alzheimer’s disease [46].

Lastly, as said before with respect to schizophrenia and autism, i.e., the two prototypical clinical populations presenting ToM deficits, structural and functional prefrontal alterations have been associated with reduced ToM performance. To go into more detail, in a voxel-based morphometry study, Hirao et al. (2008) [46] found a significant gray matter reduction in the superior frontal gyrus and left middle frontal gyrus, along with the ventromedial prefrontal cortex, the anterior cingulate cortex, the right superior temporal gyrus and the right insula, in schizophrenic patients who performed the RMET task.

In our study, MCI–AD patients performed worse than HC, a result that could be in line with a later stage of the AD course. However, Yildirim et al. (2020) [47] investigated affective ToM performance in different stages of AD (i.e., AD dementia, MCI) and subjective cognitive impairment using both the Faux Pas Recognition Test (FPR) and the RMET. They found that, while AD patients performed worse in both tasks, MCI patients showed a poor performance only in the RMET, suggesting that the ability of decoding emotion is already impaired at the early stages of AD. This ability very likely relies upon brain areas subserving the “social network”, such as the STS and the inferior frontal gyrus [48]. As for bvFTD patients, lower scores could be explained by the frontal involvement in both ToM and the underlying neuropathology.

We then evaluated the relative importance of the different areas included in the ToM network. Indeed, while in motor and primary sensory areas it is possible to draw a functional hierarchy of regions, current evidence is scant regarding its generalizability to mainly cognitive networks. To this aim, we used the connectionist paradigm to classify the regions included in the ToM network between hubs and spokes, based on their structural connectivity [49]. While published data suggest a different functional relevance of spoke and hub regions, the impact of hub damage on spoke region functional relevance (and vice versa) is poorly understood.

Here, we showed that the impact of spoke region damage on RMET performance was mediated by the integrity of hubs, while the impact of hub damage on RMET performance was independent from the integrity of spokes. The identified hubs were the bilateral superior frontal gyri in bvFTD and the left middle frontal gyrus in MCI–AD, in line with the aforementioned roles played by the PFC in ToM [50].

Indeed, in previous studies, different areas presenting strong structural connections with the PFC have been associated with RMET performance, pointing to the relevance of the network-based approach to the localization of cognitive functions. These regions include the superior temporal pole [51], the basal ganglia [52] and the posterior temporal areas [53] as assessed with both structural and functional imaging techniques. In line with this, the white matter connecting these regions [54] has also all been associated with ToM in clinical populations.

Brain hubs are thought to play a key role in information integration in neural networks as shown by the impact of hub focal lesions on network functional dynamics [55,56,57].

## 5. Conclusions

This study represents a proof-of-concept investigation of the relative importance of hub and spoke integrity on ToM abilities in MCI–AD and bvFTD patients. These pathologies represent good experimental models to study connectivity integrity because of their different patterns of damage (i.e., one more posterior and the other more anterior), and thus allow us to untangle the impact of regional damage and structural connectivity on ToM ability.

## 6. Limitations and Future Directions

A major limitation of our study is represented by its lack of longitudinal data, which may have been helpful to better understand the development of ToM deficits as neurodegeneration proceeds. Regarding the assessment of structural connectivity, we used an atlas-based approach as opposed to identifying white matter connectivity directly in our patients using diffusion MRI data. However, we focused on relatively large gray matter regions, which present a well-described and stable pattern of white matter connections. Moreover, as we focused on a network composed of a small number of regions, we used a relatively loose definition of hub (i.e., the highest connected region) as opposed to the stringent definition based on graph theory.

Moreover, while different tests have been proposed to probe ToM, here we focused only on the RMET, which assesses the affective facets of ToM, and thus caution is needed when generalizing these findings to the other components of ToM.

Our findings support the hypothesis that a key role is played by the hubs in ToM deficits, thus suggesting that network hubs could represent a final common pathway leading from regional damage to clinical deficits. We believe this study may be relevant regarding the relationship between underling structural connectivity and brain function, not only regarding social cognition, but also other neuropsychological domains. Looking forward, we thus plan to verify these findings in other neurological conditions, including stroke, and also to broaden our scope to study the neural networks underlying memory and executive functions. To this aim, it will be necessary to clearly define the components of the different networks and to choose which metrics to use to explore their integrity (i.e., using a multi-modal, multi-parametric approach to study network integrity). Potentially, this approach will allow us to characterize not only which network components are more relevant for a given cognitive function, but also which pathological mechanisms are more critical to the development of clinically relevant deficits.

## Figures and Tables

**Table 1:** Demographic and clinical features of bvFTD and MCI–AD patients. Values are shown as mean ± standard deviation.

	bvFTD (n = 24)	MCI–AD (n = 25)	HC (n = 40)	*p*-Value
Age (yr)	75.58 ± 7.66	80.72 ± 5.61	78.5 ± 9.4	*p* = 0.09
Education (yr)	9.86 ± 4.66	10.32 ± 4.07	9.9 ± 3.2	*p* = 0.714
Gender (M:F)	6:18	9:16	15:25	*p* = 0.568
MMSE score	26.41 ± 2.84	26.13 ± 3.01	28.0 ± 3.2	*p* = 0.031
RMET total score	16.25 ± 4.89	17.6 ± 6.8	28.9 ± 2.1	*p* = 0.000
GDS-15 score	3.78 ± 2.69	3.86 ± 2.79	2.0 ± 3.0	*p* = 0.014

Note: bvFTD = behavioral variant of frontotemporal dementia; MCI–AD = mild cognitive impairment due to Alzheimer’s disease; HC = healthy controls; M = male; F = female; MMSE = mini–mental state examination; RMET = Reading the Mind in the Eyes test; GDS-15 score = 15-item Geriatric Depression Scale; n.s = not significant.

**Table 2:** Connectivity matrix between RMET ROIs, considering the distribution of regional hypometabolism in the bvFTD group. Legend: Lack of structural connections are reported with “-“. Presence of structural connections between two hypometabolic areas are reported with “X”. Presence of structural connections between pair of regions including at least one without relative hypometabolism are reported with “*”.

bvFTD														
	Connection													Connections among Hypometabolic Areas in bvFTD
LMTG (*)		-	-	*	*	-	*	-	-	-	-	-	-	0
LCG (X)	-		-	-	-	X	-	-	-	-	-	-	-	1
LIFG (X)	-	-		X	X	X	-	-	-	-	-	-	-	3
LMFG (X)	*	-	X		X	X	-	-	-	-	X	X	-	5
LPG (X)	*	-	X	X		X	-	-	-	-	-	X	-	4
LSFG (X)	-	X	X	X	X		-	-	-	-	X	X	-	6
LSTG (X)	*	-	-	-	-	-		-	-	-	-	-	-	0
RMTG (*)	-	-	-	-	-	-	-		-	-	X	-	X	0
RCG (X)	-	-	-	-	-	-	-	-		-	-	X	-	1
RIFG (X)	-	-	-	-	-	-	-	-	-		X	X	-	2
RMFG (X)	-	-	-	X	-	X	-	*	-	X		X	-	4
RSFG (X)	-	-	-	X	X	X	-	-	X	X	X		-	6
RSTG (X)	-	-	-	-	-	-	-	*	-	-	-	-		0
	LMTG (*)	LCG	LIFG	LMFG	LPG	LSFG	LSTG	RMTG (*)	RCG	RIFG	RMFG	RSFG	RSTG	

Note: bvFTD = behavioral variant of frontotemporal dementia; LMTG, RMTG = left and right middle temporal gyrus; LSTG, RSTG = left and right superior temporal gyrus; LCG, RCG = left and right cingulate gyrus; LSFG, RSFG = left and right superior frontal gyrus; LIFG, RIFG = left and right inferior frontal gyrus; LMFG, RMFG = left and right middle frontal gyrus; LPG = left precentral gyrus.

**Table 3:** Connectivity matrix between RMET ROIs considering the distribution of regional hypometabolism in the MCI–AD group. Legend: Lack of structural connections are reported with “-“. Presence of structural connections between two hypometabolic areas are reported with “X”. Presence of structural connections between pair of regions including at least one without relative hypometabolism are reported with “*”.

MCI–AD														
	Connection													Connections among Hypometabolic Areas in MCI–AD
LMTG (X)		-	-	X	*	-	*	-	-	-	-	-	-	1
LCG (*)	-		-	-	-	*	-	-	-	-	-	-	-	0
LIFG (*)	-	-		*	*	*	-	-	-	-	-	-	-	0
LMFG (X)	X	-	*		*	X	-	-	-	-	*	*	-	2
LPG (*)	*	-	*	*		*	-	-	-	-	-	*	-	0
LSFG (X)	-	*	*	X	*		-	-	-	-	*	*	-	1
LSTG (*)	*	-	-	-	-	-		-	-	-	-	-	-	0
RMTG (*)	-	-	-	-	-	-	-				*	-	*	0
RCG (X)	-	-	-	-	-	-	-	-				*	-	0
RIFG (*)	-	-	-	-	-	-	-	-	-		*	*	-	0
RMFG (*)	-	-	-	*		*	-	*	-	*		*	-	0
RSFG (*)	-	-	-	*	*	*	-	-	*	*	*		-	0
RSTG (X)	-	-	-	-	-	-	-	*	-	-	-	-		0
	LMTG	LCG (*)	LIFG (*)	LMFG (X)	LPG (*)	LSFG (X)	LSTG (*)	RMTG (*)	RCG (X)	RIFG (*)	RMFG (*)	RSFG (*)	RSTG (X)	

Note: MCI–AD = mild cognitive impairment due to Alzheimer’s disease; LMTG, RMTG = left and right middle temporal gyrus; LSTG, RSTG = left and right superior temporal gyrus; LCG, RCG = left and right cingulate gyrus; LSFG, RSFG = left and right superior frontal gyrus; LIFG, RIFG = left and right inferior frontal gyrus; LMFG, RMFG = left and right middle frontal gyrus; LPG = left precentral gyrus.

**Table 4:** Correlation between RMET score and cortical RMET ROIs metabolism, corrected for age, education and MMSE score. Hub regions are reported in bold; spoke regions are reported in *italics*.

	bvFTD		MCI–AD	
	r Values	*p* Values (FDR Corrected)	r Values	*p* Values (FDR Corrected)
LMTG	r = 0.278	*p* = 0.280	*r = 0.43*	*p = 0.033*
LCG	*r = 0.48*	*p = 0.033*	r = −0.358	*p* = 0.150
LIFG	*r = 0.53*	*p = 0.010*	r = −0.027	*p* = 0.906
LMFG	*r = 0.65*	*p = 0.004*	**r = 0.69**	***p* = 0.01**
LPG	*r = 0.56*	*p = 0.005*	r = 0.010	*p* = 0.966
LSFG	**r = 0.72**	***p* = 0.004**	*r = 0.50*	*p = 0.039*
LSTG	r = −0.205	*p* = 0.431	r = −0.190	*p* = 0.398
RMTG	r = −0.101	*p* = 0.699	r = 0.078	*p* = 0.729
RCG	*r = 0.45*	*p = 0.032*	r = −0.478	*p* = 0.120
RIFG	*r = 0.52*	*p = 0.013*	r = 0.064	*p* = 0.776
RMFG	*r = 0.57*	*p = 0.005*	r = 0.196	*p* = 0.383
RSFG	**r = 0.70**	***p* = 0.004**	r = 0.086	*p* = 0.703
RSTG	r = 0.226	*p* = 0.382	r = −0.241	*p* = 0.281

Note: bvFTD = behavioral variant of frontotemporal dementia; MCI–AD = mild cognitive impairment due to Alzheimer’s disease; n.s = not significant; MMSE = mini–mental state examination; LMTG, RMTG = left and right middle temporal gyrus; LSTG, RSTG = left and right superior temporal gyrus; LCG, RCG = left and right cingulate gyrus; LSFG, RSFG = left and right superior frontal gyrus; LIFG, RIFG = left and right inferior frontal gyrus; LMFG, RMFG = left and right middle frontal gyrus; LPG = left precentral gyrus.

## Data Availability

Data available to investigator upon reasonable request for scientific collaboration.

## Supplementary Materials

The following supporting information can be downloaded at: https://www.mdpi.com/article/10.3390/biomedicines10030544/s1, Table S1: Areas of relative hypometabolism in bvFTD group (BA = Brodmann Area); Table S2: Areas of relative hypometabolism in MCI-AD group (BA = Brodmann Area).

## References

[1] Baron-Cohen S., Campbell R., Karmiloff-Smith A., Grant J., Walker J. (1995). Are children with autism blind to the mentalistic significance of the eyes?. Br. J. Dev. Psychol..

[2] Orso B., Arnaldi D., Famà F., Girtler N., Brugnolo A., Doglione E., Filippi L., Massa F., Peira E., Bauckneht M. (2020). Anatomical and neurochemical bases of theory of mind in de novo Parkinson’s Disease. Cortex.

[3] Pardini M., Gialloreti L.E., Mascolo M., Benassi F., Abate L., Guida S., Viani E., Monte O.D., Schintu S., Krueger F. (2013). Isolated theory of mind deficits and risk for frontotemporal dementia: A longitudinal pilot study. J. Neurol. Neurosurg. Psychiatry.

[4] Schurz M., Radua J., Aichhorn M., Richlan F., Perner J. (2014). Fractionating theory of mind: A meta-analysis of functional brain imaging studies. Neurosci. Biobehav. Rev..

[5] Schurz M., Perner J. (2015). An evaluation of neurocognitive models of theory of mind. Front. Psychol..

[6] Zeng Y., Zhao Y., Zhang T., Zhao D., Zhao F., Lu E. (2020). A Brain-Inspired Model of Theory of Mind. Front. Neurorobotics.

[7] Ricci M., Cimini A., Camedda R., Chiaravalloti A., Schillaci O. (2021). Tau Biomarkers in Dementia: Positron Emission Tomography Radiopharmaceuticals in Tauopathy Assessment and Future Perspective. Int. J. Mol. Sci..

[8] Chiaravalloti A., Barbagallo G., Ricci M., Martorana A., Ursini F., Sannino P., Karalis G., Schillaci O. (2018). Brain metabolic correlates of CSF Tau protein in a large cohort of Alzheimer’s disease patients: A CSF and FDG PET study. Brain Res..

[9] Balogh L., Tanaka M., Török N., Vécsei L., Taguchi S. (2021). Crosstalk between Existential Phenomenological Psychotherapy and Neurological Sciences in Mood and Anxiety Disorders. Biomedicines.

[10] Buckner R.L., Andrews-Hanna E.J.R., Schactera D.L. (2008). The Brain’s Default Network: Anatomy, Function, and Relevance to Disease. Ann. N. Y. Acad. Sci..

[11] Kyeong S., Kim J., Kim J., Kim E.J., Kim H.E., Kim J.-J. (2020). Differences in the modulation of functional connectivity by self-talk tasks between people with low and high life satisfaction. NeuroImage.

[12] Cai S., Peng Y., Chong T., Zhang Y., Von Deneen K.M., Huang L., Cai Y.P.S. (2017). Differentiated effective connectivity patterns of the executive control network in progressive MCI: A potential biomarker for predicting AD. Curr. Alzheimer Res..

[13] Zhao Q., Lu H., Metmer H., Li W.X., Lu J. (2018). Evaluating functional connectivity of executive control network and frontoparietal network in Alzheimer’s disease. Brain Res..

[14] Cieri F., Esposito R., Cera N., Pieramico V., Tartaro A., Di Giannantonio M. (2017). Late-Life Depression: Modifications of Brain Resting State Activity. J. Geriatr. Psychiatry Neurol..

[15] Seeley W.W. (2019). The Salience Network: A Neural System for Perceiving and Responding to Homeostatic Demands. J. Neurosci..

[16] Dai L., Zhou H., Xu X., Zuo Z. (2019). Brain structural and functional changes in patients with major depressive disorder: A literature review. PeerJ.

[17] Battaglia S., Harrison B.J., Fullana M.A. (2021). Does the human ventromedial prefrontal cortex support fear learning, fear extinction or both? A commentary on subregional contributions. Mol. Psychiatry.

[18] Van Den Heuvel M.P., Stam C.J., Kahn R.S., Pol H.E.H. (2009). Efficiency of functional brain networks and intellectual performance. J. Neurosci..

[19] Hwang K., Hallquist M.N., Luna B. (2013). The development of hub architecture in the human functional brain network. Cereb. Cortex.

[20] Albert R., Jeong H., Barabási A.L. (2000). Error and attack tolerance of complex networks. Nature.

[21] Aben H.P., Biessels G.J., Weaver N.A., Spikman J.M., Visser-Meily J.M., de Kort P.L., Reijmer Y.D., PROCRAS Study Group (2019). Extent to Which Network Hubs Are Affected by Ischemic Stroke Predicts Cognitive Recovery. Stroke.

[22] Albert M.S., DeKosky S.T., Dickson D., Dubois B., Feldman H.H., Fox N.C., Gamst A., Holtzman D.M., Jagust W.J., Petersen R.C. (2013). The Diagnosis of Mild Cognitive Impairment due to Alzheimer’s Disease: Recommendations from the National Institute on Aging-Alzheimer’s Association Workgroups on Diagnostic Guidelines for Alzheimer’s Disease. Focus.

[23] Jack C.R., Bennett D.A., Blennow K., Carrillo M.C., Dunn B., Haeberlein S.B., Holtzman D.M., Jagust W., Jessen F., Karlawish J. (2018). NIA-AA Research Framework: Toward a biological definition of Alzheimer’s disease. Alzheimer Dement..

[24] Rascovsky K., Hodges J.R., Kipps C., Johnson J.K., Seeley W.W., Mendez M.F., Knopman D.S., Kertesz A., Mesulam M.M., Salmon D.P. (2007). Diagnostic Criteria for the Behavioral Variant of Frontotemporal Dementia (bvFTD): Current Limitations and Future Directions. Alzheimer Dis. Assoc. Disord..

[25] Baron-Cohen S., Wheelwright S., Hill J., Raste Y., Plumb I. (2001). The “Reading the Mind in the Eyes” Test revised version: A study with normal adults, and adults with Asperger syndrome or high-functioning autism. J. Child Psychol. Psychiatry.

[26] Harkness K.L., Sabbagh M.A., Jacobson J.A., Chowdrey N.K., Chen T. (2005). Enhanced accuracy of mental state decoding in dysphoric college students. Cogn. Emot..

[27] Pardini M., Nichelli P.F. (2009). Age-related decline in mentalizing skills across adult life span. Exp. Aging Res..

[28] Varrone A., Asenbaum S., Borght T.V., Booij J., Nobili F., Någren K., Darcourt J., Kapucu Ö.L., Tatsch K., Bartenstein P. (2009). EANM procedure guidelines for PET brain imaging using [^18^F] FDG, version 2. Eur. J. Nucl. Med. Mol. Imaging.

[29] Della Rosa P.A., Cerami C., Gallivanone F., Prestia A., Caroli A., Castiglioni I., Gilardi M.C., Frisoni G., Friston K., the EADC-PET Consortium (2014). A Standardized [^18^F]-FDG-PET Template for Spatial Normalization in Statistical Parametric Mapping of Dementia. Neuroinformatics.

[30] Molenberghs P., Johnson H., Henry J.D., Mattingley J.B. (2016). Understanding the minds of others: A neuroimaging meta-analysis. Neurosci. Biobehav. Rev..

[31] Abu-Akel A. (2003). A neurobiological mapping of theory of mind. Brain Res. Rev..

[32] Cotter J., Granger K., Backx R., Hobbs M., Looi C.Y., Barnett J.H. (2018). Social cognitive dysfunction as a clinical marker: A systematic review of meta-analyses across 30 clinical conditions. Neurosci. Biobehav. Rev..

[33] Koff E., Brownell H., Winner E., Albert M., Zaitchik D. (2004). Inference of mental states in patients with Alzheimer’s disease. Cogn. Neuropsychiatry.

[34] De Lucena A.T., Bhalla R.K., Dos Santos T.T.B.A., Dourado M.C.N. (2020). The relationship between theory of mind and cognition in Alzheimer’s disease: A systematic review. J. Clin. Exp. Neuropsychol..

[35] Orso B., Mattei C., Arnaldi D., Massa F., Serafini G., Plantone D., Doglione E., Grafman J., Nobili F., Pardini M. (2020). Clinical and MRI Predictors of Conversion From Mild Behavioural Impairment to Dementia. Am. J. Geriatr. Psychiatry.

[36] Dal Monte O., Schintu S., Pardini M., Berti A., Wassermann E.M., Grafman J., Krueger F. (2014). The left inferior frontal gyrus is crucial for reading the mind in the eyes: Brain lesion evidence. Cortex.

[37] Rowe A.D., Bullock P.R., Polkey C.E., Morris R.G. (2001). ‘Theory of mind’ impairments and their relationship to executive functioning following frontal lobe excisions. Brain.

[38] Le Bouc R., Lenfant P., Delbeuck X., Ravasi L., Lebert F., Semah F., Pasquier F. (2012). My belief or yours? Differential theory of mind deficits in frontotemporal dementia and Alzheimer’s disease. Brain.

[39] Ellena G., Battaglia S., Làdavas E. (2020). The spatial effect of fearful faces in the autonomic response. Exp. Brain Res..

[40] Borgomaneri S., Vitale F., Battaglia S., Avenanti A. (2021). Early Right Motor Cortex Response to Happy and Fearful Facial Expressions: A TMS Motor-Evoked Potential Study. Brain Sci..

[41] Kim J., Kim Y.-K. (2021). Crosstalk between Depression and Dementia with Resting-State fMRI Studies and Its Relationship with Cognitive Functioning. Biomedicines.

[42] Tanaka M., Vécsei L. (2021). Editorial of Special Issue “Crosstalk between Depression, Anxiety, and Dementia: Comorbidity in Behavioral Neurology and Neuropsychiatry”. Biomedicines.

[43] Bryll A., Krzyściak W., Karcz P., Pilecki M., Śmierciak N., Szwajca M., Skalniak A., Popiela T. (2021). Determinants of Schizophrenia Endophenotypes Based on Neuroimaging and Biochemical Parameters. Biomedicines.

[44] Ji G., Li S., Ye L., Guan J. (2021). Gene Module Analysis Reveals Cell-Type Specificity and Potential Target Genes in Autism’s Pathogenesis. Biomedicines.

[45] Chiarelli A.M., Perpetuini D., Croce P., Filippini C., Cardone D., Rotunno L., Anzoletti N., Zito M., Zappasodi F., Merla A. (2021). Evidence of Neurovascular Un-Coupling in Mild Alzheimer’s Disease through Multimodal EEG-fNIRS and Multivariate Analysis of Resting-State Data. Biomedicines.

[46] Hirao K., Miyata J., Fujiwara H., Yamada M., Namiki C., Shimizu M., Sawamoto N., Fukuyama H., Hayashi T., Murai T. (2008). Theory of mind and frontal lobe pathology in schizophrenia: A voxel-based morphometry study. Schizophr. Res..

[47] Yildirim E., Buyukiscan E.S., Demirtas-Tatlidede A., Bilgiç B., Gurvit H. (2020). An investigation of affective theory of mind ability and its relation to neuropsychological functions in Alzheimer’s disease. J. Neuropsychol..

[48] Overgaauw S., Van Duijvenvoorde A.C.K., Moor B.G., Crone E. (2014). A longitudinal analysis of neural regions involved in reading the mind in the eyes. Soc. Cogn. Affect. Neurosci..

[49] Van den Heuvel M.P., Sporns O. (2013). Network hubs in the human brain. Trends Cogn. Sci..

[50] Abu-Akel A., Shamay-Tsoory S. (2011). Neuroanatomical and neurochemical bases of theory of mind. Neuropsychologia.

[51] Irish M., Hodges J.R., Piguet O. (2014). Right anterior temporal lobe dysfunction underlies theory of mind impairments in semantic dementia. Brain.

[52] Bodden M.E., Dodel R., Kalbe E. (2009). Theory of mind in Parkinson’s disease and related basal ganglia disorders: A systematic review. Mov. Disord..

[53] Brunet E., Sarfati Y., Hardy-Baylé M.-C., Decety J. (2003). Abnormalities of brain function during a nonverbal theory of mind task in schizophrenia. Neuropsychologia.

[54] Isernia S., Cabinio M., Pirastru A., Mendozzi L., Di Dio C., Marchetti A., Massaro D., Baglio F. (2020). Theory of mind network in multiple Sclerosis: A double disconnection mechanism. Soc. Neurosci..

[55] Honey C.J., Sporns O. (2008). Dynamical consequences of lesions in cortical networks. Hum. Brain Mapp..

[56] Foulon C., Cerliani L., Kinkingnéhun S., Levy R., Rosso C., Urbanski M., Volle E., De Schotten M.T. (2018). Advanced lesion symptom mapping analyses and implementation as BCBtoolkit. GigaScience.

[57] Pardini M., Yaldizli Ö., Sethi V., Muhlert N., Liu Z., Samson R.S., Altmann D.R., Ron M.A., Wheeler-Kingshott C.A., Miller D.H. (2015). Motor network efficiency and disability in multiple sclerosis. Neurology.

