# Supplementary material for: The Role of Hub and Spoke Regions in Theory of Mind in Early Alzheimer’s Disease and Frontotemporal Dementia
Source: Biomedicines. 2022 Feb 24;10(3):544. doi: 10.3390/biomedicines10030544 (PMC8945345; doi:10.3390/biomedicines10030544)
Supplement: Supplementary file 1 [file biomedicines-10-00544-s001.zip › biomedicines-1437838-supplementary.pdf]

Supplementary Table S1: Areas of relative hypometabolism in bvFTD group (BA = Brodmann Area).

N. of voxel	Coordinates			Laterality	Anatomic Area	BA	T	Z
	X	Y	Z					
30259	11.96	9.35	5.36	Right	Caudate	Caudate Head	8.50	6.86
	−12.09	11.33	5.15	Left	Caudate	Caudate Head	8.20	6.69
	−6.15	28.82	−20.12	Left	Rectal Gyrus	11	7.87	6.50
	−36.15	11.46	4.75	Left	Insula	13	7.62	6.35
	−43.08	7.7	−33.56	Left	Middle Temporal Gyrus	21	7.48	6.26
	12.16	60.17	4.78	Right	Medial Frontal Gyrus	10	7.35	6.18
	−19.3	58.47	4.09	Left	Medial Frontal Gyrus	10	7.34	6.17
	8.5	58.68	0.97	Right	Medial Frontal Gyrus	10	7.25	6.12
	6.7	57.17	−2.8	Right	Superior Frontal Gyrus	10	7.12	6.04
	−31.96	13.23	−32.85	Left	Superior Temporal Gyrus	38	6.77	5.81
	12.34	28.55	−18.03	Right	Medial Frontal Gyrus	25	6.73	5.79
	−45.25	25.42	−3.09	Left	Inferior Frontal Gyrus	47	6.67	5.75
	−6.47	57.54	13.22	Left	Medial Frontal Gyrus	10	6.66	5.74
	−47.17	26.77	2.41	Left	Inferior Frontal Gyrus	45	6.65	5.73
	−30.38	14.86	−11.04	Left	Inferior Frontal Gyrus	13	6.59	5.69
	−41.5	38.62	−3.58	Left	Middle Frontal Gyrus	47	6.58	5.69
867	9.82	19.96	31.56	Right	Cingulate Gyrus	32	5.21	4.71
	9.71	6.39	35.68	Right	Cingulate Gyrus	24	4.99	4.54
	4.07	−27.12	32.41	Right	Cingulate Gyrus	23	4.46	4.13
	5.99	−1.04	34.91	Right	Cingulate Gyrus	24	3.84	3.62
	11.91	34.56	16.76	Right	Anterior Cingulate	32	3.76	3.55
	4.08	−41.67	27.42	Right	Cingulate Gyrus	31	3.73	3.53
	10.18	37.3	7.98	Right	Anterior Cingulate	32	3.62	3.43
	11.99	36.94	11.58	Right	Anterior Cingulate	32	3.56	3.38
321	−8.67	20.23	29.47	Left	Cingulate Gyrus	32	4.91	4.48
209	−43.19	−51.57	−42.78	Left	Cerebellar Tonsil	*	4.44	4.11

380	54.11	−56.14	19.69	Right	Superior Temporal Gyrus	22	3.91	3.67
	50.26	−59.03	30.16	Right	Superior Temporal Gyrus	39	3.83	3.61
	48.44	−64.26	26.03	Right	Middle Temporal Gyrus	39	3.83	3.61

Supplementary Table S2: Areas of relative hypometabolism in MCI-AD group (BA = Brodmann Area).

N. voxel	Coordinates			Laterality	Anatomic Area	BA	T	Z
	X	Y	Z					
4162	−33.1	−70.13	31.31	Left	Middle Temporal Gyrus	39	5.89	5.25
	−47.01	−19.58	−23.6	Left	Fusiform Gyrus	20	5.12	4.67
	−46.05	−66.33	31.45	Left	Angular Gyrus	39	5.03	4.61
	−50.97	−54.32	−14.34	Left	Inferior Temporal Gyrus	20	4.90	4.50
	−39.52	−9.78	−27.95	Left	Fusiform Gyrus	20	4.84	4.46
	−58.26	−27.85	−15.56	Left	Middle Temporal Gyrus	21	4.83	4.44
	−56.35	−25.65	−18.92	Left	Inferior Temporal Gyrus	20	4.79	4.42
	−33.85	−1.66	−34.29	Left	Uncus	20	4.79	4.42
	−52.72	−42.61	−18.67	Left	Inferior Temporal Gyrus	37	4.77	4.40
	−35.69	3.94	−33.79	Left	Middle Temporal Gyrus	21	4.74	4.38
	−22.85	−6.14	−27.32	Left	Uncus	36	4.65	4.31
	−37.84	−33.02	−21.11	Left	Parahippocampal Gyrus	36	4.25	3.98
	−58.41	−41.76	−7.87	Left	Middle Temporal Gyrus	21	4.00	3.76
	−53.02	−61.29	−0.62	Left	Middle Temporal Gyrus	37	3.88	3.67
	−55.09	−55.57	17.9	Left	Superior Temporal Gyrus	22	3.76	3.57
	−58.18	−9.04	−15.58	Left	Inferior Temporal Gyrus	21	3.25	3.11
1931	−3.31	−47.05	24.99	Left	Posterior Cingulate	23	5.84	5.22
	0.36	−32.69	31.82	Left	Cingulate Gyrus	31	5.63	5.06
	−7.15	−71.77	27.99	Left	Precuneus	31	4.61	4.27
	3.8	−78.47	38.35	Right	Cuneus	19	3.96	3.73
1032	40.97	−72.02	28.78	Right	Middle Temporal Gyrus	39	5.28	4.80

	50.2	−46.68	38.54	Right	Inferior Parietal Lobule	40	3.44	3.28
	48.32	−50.58	39.94	Right	Inferior Parietal Lobule	40	3.42	3.26
180	−21.44	−35.02	−1.21	Left	Parahippocampal Gyrus	27	5.19	4.73
194	−38.03	46.0	17.0	Left	Middle Frontal Gyrus	46	5.17	4.72
1702	45.63	−8.36	−26.37	Right	Fusiform Gyrus	20	5.04	4.61
	63.8	−31.04	−6.59	Right	Middle Temporal Gyrus	21	4.54	4.22
	32.78	−0.14	−33.02	Right	Uncus	20	4.52	4.20
	40.2	3.54	−32.54	Right	Middle Temporal Gyrus	21	4.43	4.12
	54.59	−48.93	−15.65	Right	Fusiform Gyrus	37	4.38	4.08
	54.7	−24.36	−16.92	Right	Inferior Temporal Gyrus	20	4.27	3.99
	56.2	−54.41	1.88	Right	Middle Temporal Gyrus	37	3.74	3.55
332	−35.03	−1.23	57.62	Left	Middle Frontal Gyrus	6	4.80	4.43
	−14.51	23.58	53.11	Left	Superior Frontal Gyrus	6	4.31	4.03
	−29.38	8.58	53.24	Left	Middle Frontal Gyrus	6	4.01	3.77
148	15.49	−30.32	7.07	Right	Thalamus	Pulvinar	4.26	3.99
